# Real-time three-dimensional transthoracic echocardiography in daily practice: initial experience

**DOI:** 10.1186/1476-7120-10-14

**Published:** 2012-03-26

**Authors:** Ashraf M Anwar, Youssef FM Nosir, Siti Khairani Zainal-Abidin, Aref Ajam, Hassan Chamsi-Pasha

**Affiliations:** 1 Department of Cardiology, King Fahd Armed Forces Hospital, P.O. Box: 9862, Jeddah 21159, Saudi-Arabia; 2 Department of Cardiology, Al-Azhar University, Cairo, Egypt; 3 Cardiology department of, King Fahd Armed Forces Hospital, Jeddah, Saudi-Arabia; 4 Cardiology Department, Pulau Pinang Hospital, Jalan Residensi, Pulau Pinang, Malaysia

## Abstract

**Aim of the work:**

To evaluate the feasibility and possible additional value of transthoracic real-time three-dimensional echocardiography (RT3D-TTE) for the assessment of cardiac structures as compared to 2D-TTE.

**Methods:**

320 patients (mean age 45 ± 8.4 years, 75% males) underwent 2D-TTE and RT3D-TTE using 3DQ-Q lab software for offline analysis. Volume quantification and functional assessment was performed in 90 patients for left ventricle and in 20 patients for right ventricle. Assessment of native (112 patients) and prosthetic (30 patients) valves morphology and functions was performed. RT3D-TTE was performed for evaluation of septal defects in 30 patients and intracardiac masses in 52 patients.

**Results:**

RT3D-TTE assessment of left ventricle was feasible and reproducible in 86% of patients while for right ventricle, it was (55%). RT3D-TTE could define the surface anatomy of mitral valve optimally (100%), while for aortic and tricuspid was (88% and 81% respectively). Valve area could be planimetered in 100% for the mitral and in 80% for the aortic. RT3D-TTE provided a comprehensive anatomical and functional evaluation of prosthetic valves. RT3D-TTE enface visualization of septal defects allowed optimal assessment of shape, size, area and number of defects and evaluated the outcome post device closure. RT3D-TTE allowed looking inside the intracardiac masses through multiple sectioning, valuable anatomical delineation and volume calculation.

**Conclusion:**

Our initial experience showed that the use of RT3D-TTE in the assessment of cardiac patients is feasible and allowed detailed anatomical and functional assessment of many cardiac disorders.

## Introduction

Conventional 2-dimensional echocardiography (2DE) has been established as the most widely diagnostic tool that helps in morphological and functional assessment of cardiac chambers and valves. Despite the advancement in technology of echo machines and its analysis software, 2DE application still carries many limitations particularly with complex anomalies and cardiac chamber quantification. It requires mental conceptualization of a series of multiple tomographic images into an imaginary multidimensional reconstruction [1]. Many of 2DE formulas used for volume quantification and ejection fraction calculation especially for left ventricle (LV) are based on geometric assumption that may not be accurate in the setting of chamber dilatation or distortion and in the presence of regional wall motion abnormalities. Interobserver variability for 2DE images interpretation is still wide due to different ways of data interpolation especially for measurement of mitral and aortic valve orifice area [2]. Imaging of the heart in 3D provides better understanding and assessment of cardiac structures in a real shape. The advanced technology of matrix array transducer improved the contrast resolution and penetration and enabled to image the entire heart by a pyramidal full-volume acquisition of four cardiac cycles. The development in 3D software made the off-line data analysis faster and easier and improved both temporal and spatial resolution of the images. We report our experience with real-time 3D-transthoracic echocardiography (RT3D-TTE) in comparison with 2D-TTE for the assessment of different cardiac conditions.

## Methods

### Study population

Among the 425 patients screened with 2D-TTE, 105 patients (24.7%) had inadequate image quality, therefore they were excluded. The remaining 320 patients with good image quality were included in the study. Patients planed for volume quantification were selected to be in sinus rhythm. To include most of variable structural heart diseases as diagnosed by 2D- TTE, a considerable sample of patients was selected for analysis by both 2D- TTE and RT3D-TTE techniques (Table 1).

**Table 1:** Clinical diagnosis of the 320 studied patients

Clinical Diagnosis	No. (%)	RT3D-TTE application
Normal subjects	35(11%)	volume quantification of LV and RV anatomical and functional assessment of MV and TV

Ischemic Heart Disease	35(11%)	volume quantification of LV and RV

Cardiomyopathies	44(13.7%)	volume quantification of LV and RV

Pulmonary hypertension	13(4.3%)	volume quantification of RV anatomical and functional assessment of TV

Rheumatic Heart Disease	50(16.6%)	anatomical and functional assessment of all valves

Prosthetic valves and rings	30(10%)	anatomical and functional assessment of all valves

Congenital heart disease		
• MVP	5(1.7%)	Define the prolapsed scallop and assess mitral regurgitation
• ASD	18(6%)	
• VSD	6(2%)	Measure number, size& rims.
• PFO	3(1%)	
• Malformed AV	25(8%)	Define cusps (number & morphology). Measure valve area
• PS	5(1.7%)	

Intracardiac masses and thrombi	52(17.3%)	Anatomical delineation and volume calculation

### Transthoracic 2DE examination

2D-TTE was performed in all subjects with Philips Sonos 7500 with 3.5 MHz probe and Philips IE-33 with S5-1 probe. Examination was performed while the patient in the left lateral decubitus position using the standard apical, parasternal and subcostal views to obtain all quantitative and qualitative complete study according to American Society of Echocardiography guidelines [3]. Adequacy for the qualitative analysis was scored and classified according to a subjective 3-point scale for image quality (1 = not visualized, 2 = fair, and 3 = good).

### Transthoracic RT3D-TTE examination

RT3D-TTE was performed with the same machines using X4 and X3-1 matrix array transducers. In each patient, only 1 cardiac structure was selected for acquisition and analysis (focused and complete study). Data acquisition was based on recommendations of Adhoc 3D Echo Protocol Working Group endorsed by the International Society of Cardiovascular Ultrasound [4]. Adjustment of gain and brightness were performed with XRes Adaptive Image Processing (provides image enhancement to reduce speckle, haze, and clutter artifacts, and enhances edges) set to low to improve delineation of anatomic structures. Vision H (color map to enhance depth perception by coding nearer structures orange and structures further away blue) was used to improve visualization of the targeted cardiac structure. For full volume, acquisition of 4-7 consecutive beats was performed with optimization of sector size, volume size to get the optimal frame rate (> 30 mHz). Zoom function was used for clear visualization of valve leaflets and septal defects. The 3D datasets were stored digitally and then transferred to an offline analysis using (3DQ-Q lab) software to be analyzed by two expert cardiologists (Anwar AM and Nosir YF).

#### Qualitative analysis

The acquired 3D images (Live 3D and full volume) were analyzed by (3DQ-Q lab) software system. The region of interest was obtained using the crop function by the different cut planes in various directions and angles. Setting of gain, contrast and brightness was adjusted to obtain an optimal image quality required for the analysis of relevant features of each target. Adequacy of the qualitative analysis for 3D images was scored as performed for 2D images

#### Chamber quantification

**Figure 1:**
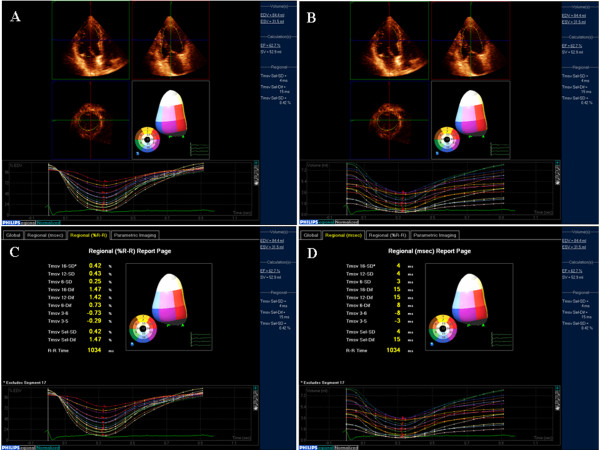
**RT3D-TTE calculation of global and regional LV systolic function**.

Quantitative analysis was done using dedicated software analysis systems (QLAB Advanced Quantification software, version 7.0, Philips Ultrasound, USA). To avoid stitching artifacts due to arrhythmia, all patients included for volume quantification were in sinus rhythm. Full volume image acquisition was used to calculate both global and segmental LV volumes and ejection fraction (EF). Through segmentation of LV cast into 16-segment model (six basal, six mid-segments and four apical segments) and estimation of time-volume curve, the systolic dyssynchrony was defined (Figure 1). Systolic dyssynchrony index was derived based on the standard deviation of mean time-to-minimal regional volume of the 16 LV segments during a cardiac cycle [5].

Right ventricular (RV) volume and EF analysis were performed using the software analysis system (3DQ-Q lab) software. The images were obtained from apical window with modification of the probe positions to get the entire RV into the 3D box. The images were cropped with manual tracing of its endocardial borders in eight contiguous volumetric slices. Then, the end-systolic volume, end-diastolic volume and EF were calculated. RT3D-TTE measurements were validated against the measurement obtained by cardiac MRI in 32 patients (25 for LV and 7 for RV).

Volume quantification of the intracardiac masses was performed using (3DQ-Q lab) software. The images were selected for analysis depending on the optimal visualization regardless of the acoustic window and/or the probe position.

#### Qualitative and quantitative assessment of valves

The qualitative assessment of valve leaflets included mobility, thickness and calcification were assessed by live 3D, zoom and full volume images. Valve area was defined as the narrowest orifice at the time of maximal valve opening and measured through off-line analysis of the acquired full volume images

#### Assessment of ventricular and atrial septal defects (ASD and VSD)

2D-TTE, 2D-TEE and RT3D-TTE were performed for all patients with suspicious diagnosis of ASD (history, clinical, ECG or chest X ray). If the diagnosis of ASD was established, the assessment of its shape, number, and size were compared between the three techniques. In patients enrolled for transcatheter device closure, the balloon occlusive diameter was used as the gold standard for comparison between the three techniques. Assessment of VSD by both 2D-TTE and RT3D-TTE techniques was performed and data were compared.

### Cardiac magnetic resonance imaging (CMR)

CMR was performed in 25 patients for volumetric quantification of both LV and RV. CMR was requested for those patients to assess viability before revascularization. After localization of the heart using three-plane and oblique survey images, 10-12 contiguous short-axis cine images were prescribed to cover the entire LV from base to apex. Images were acquired using a multislice cine vectocardiographic gated balanced fast-field echo sequence with SENSE during serial breath-holds. Twenty cine phases were acquired using retrospective gating with a temporal resolution varying between 25-50 ms. Slice thickness was 8 mm and slice spacing was 2 mm. In every end-diastolic and end-systolic short-axis slice, endocardial and epicardial contours were manually traced to calculate volumes and systolic function.

### Statistical analysis

All quantitative data obtained by 2D-TTE and RT3D-TTE were presented as mean ± SD. To determine whether the difference in values between both techniques was statistically significant or not, a paired sample t-test was performed. The level of significance was set to *P *< 0.05. Interobserver and intraobserver variabilities for 2D-TTE and RT3D-TTE measurements in all patients were calculated according to the Bland and Altman method [6]. Interobsever and intraobserver agreements for qualitative analysis score by 2D-TTE and RT3D-TTE were calculated using kappa values and classified as excellent with value of 0.93-1.0, very good 0.81-0.92, good 0.41-0.60, and poor ≤ 0.4 [7].

## Results

A total of 320 patients (mean age 45 ± 8.4 years, 75% males) were enrolled in this study for evaluation of their cardiac condition using RT3D-TTE. The number of patients in each group of cardiac abnormalities was depending on the referral rate. The average time required for 3D data acquisition was 3.2 ± 1.5 min. Data analysis by Q-lab software was widely varied (4.5 to 15 min) according to the target structure.

### LV analysis

LV assessment of 90 cases with different diagnosis (15 normals, 35 patients with different types of cardiomyopathy, 35 patients with ischemic heart disease, and 5 patients with adult congenital heart disease) was performed by both 2D-TTE and RT3D-TTE. Visualization adequacy by both techniques was good in 51 cases (57%), and fair in 26 cases (29%). LV endocardial borders were not visualized in 13 cases (14%), and therefore excluded from the analysis. Interobserver and intraobserver agreement for adequacy of analysis by both techniques were very good (kappa: 0.81 and 0.83 for 2D-TTE) and (0.85 and 0.87 for RT3D-TTE). Complete offline analysis using 3DQ-Advanced software required 3-5 minutes.

The RT3D-TTE measurements of end-diastolic, end-systolic volumes and ejection fraction were well correlated with 2D-TTE measurements (r = 0.94) with significant underestimation of end-systolic volume by 2D-TTE (*p *= 0.01). RT3D-TTE measurement correlated better with CMR than with 2D-TTE (r = 0.95 vs 0.88). Compared to CMR, all 2D-TTE measurements and end-systolic volume by RT3D-TTE were significantly underestimated. However, the mean difference by Bland& Altman method between CMR and both 2D-TTE and RT3D-TTE measurement showed no significant difference (Table 2).

**Table 2:** Volumetric quantification of both ventricles by 2D-TTE, RT3D-TTE and CMR

	2D-TTE	CMR	RT3D-TTE	*P *value		
				*	**$**	**£**

LV end-diastolic (mL)	126.6 ± 40.9	167.2 ± 65.3	143.9 ± 45.7	0.005	0.09	0.06

Mean difference	(62.2 ± 37.9)	(61.2 ± 46.2)				

LV end-systolic (mL)	89.7 ± 38.4	108.0 ± 65.9	97.1 ± 46.6	0.0001	0.01	0.02

Mean difference	(45.0 ± 31.6)	(47.5 ± 32.9)				

LV ejection fraction (%)	29.4 ± 0.8	35.3 ± 0.5	32.2 ± 0.4			

RV end-diastolic (mL)	107.2 ± 30.5	135.2 ± 40.3	125.1 ± 35.4	0.0001	0.07	0.14

RV end-systolic (mL)	52.6 ± 17. 5	68.1 ± 25.8	64.7 ± 23. 6	0.0001	0.40	0.19

RV ejection fraction (%)	51. 4 ± 40.6	49.6 ± 35.2	48.8 ± 30. 9			

*: *P *value between 2D-TTE and CMR

$: *P *value between 2D-TTE and RT3D-TTE

£: *P *value between RT3D-TTE and CMR

RT3D-TTE assessment of LV synchronization was performed in 15 patients scheduled for cardiac resynchronization therapy (CRT) according to European society of cardiology guideline [8]. The time-volume curves were obtained for the 16-segments before CRT to identify the dyssynchrony index and dyssynchronous segments. In 12 patients, RT3D-TTE detected interventricular and intraventricular dys-synchrony while in 3 patients; no dyssynchrony was detected. RT3D-TTE was repeated for those patients after CRT implant to assess the immediate result. The RT3D-TTE time volume curves became synchronized in the 12 patients who had dys-synchrony before CRT.

### RV analysis

Assessment of RV shape, volume, and function was performed in 20 cases (5 normals, 6 patients with pulmonary hypertension, 5 patients with RV dysplasia and 4 patients with dilated cardiomyopathy). Adequate visualization and complete analysis were obtained in 11 cases (55%), (5 normals, 3 patients with RV dysplasia and 3 patients with pulmonary hypertension). In the remaining 9 patients (45%), the analysis was not completed because the sector width of full volume 3D images could not include the whole dilated RV. Both 2D-TTE and RT3D-TTE measurements of RV volumes were well correlated (r = 0.87) but the RT3D-TTE values were higher than by 2D-TTE. CMR was performed in 7 patients for comparison, 2D-TTE measurements were significantly underestimated (Table 2). RT3D-TTE values had better correlation with CMR measurements than 2D-TTE (r = 0.86 vs 0.91)

### Valvular heart disease

#### Mitral valve (MV)

**Figure 2:**
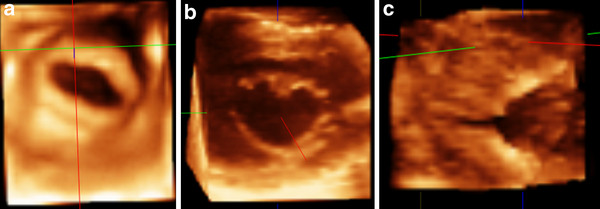
**Example of patient with mitral stenosis showing the stenotic valve orifice before (A) and after balloon valvuloplasty (B)**. (C) Side view of partially splitted commissure.

Forty patients (20 with rheumatic mitral stenosis, 10 with mitral regurgitation, 5 with mitral valve prolapse and 5 normals) were studied. Adequate visualization was achieved in all patients. In patients with rheumatic mitral stenosis, both 2D-TTE and RT3D-TTE measurement of MV area were comparable (0.91 ± 0.13 cm^2 ^vs. 0.92 ± 0.14 cm^2^). RT3D-TTE provided more information about leaflets mobility, thickness, distribution and extent of calcification. Assessment of both commissures was clearly obtained by RT3D-TTE. Assessment of balloon mitral commissurotomy results in 6 patients by RT3D-TTE showed its ability to visualize and assess the degree of commissural splitting in the en face view with rotation of image from right to left (swivel mode) (Figure 2). By 2D-TTE, it was essential to modify the probe position to achieve visualization of each MV commissure separately because both commissures are not at the same level

In 10 patients with mitral regurgitation, it was possible to assess the annulus size and function through cropping of the full 3D volume. The assessment of regurgitant jet (s) was performed by color flow RT3D-TTE. It was possible to visualize the shape and size of vena contracta in all 10 patients. Different shapes of vena contracta were found e.g. oval, circular, and irregular. Calculation of regurgitant jet volume was obtained only in 4 patients with central regurgitation. In the remaining 6 patients, the regurgitant volume could not be obtained because the jet was eccentric and directed posteriorly in 4 patients and multiple in 3 patients.

In 5 patients with MV prolapse, The RT3D-TTE enface view from its atrial and ventricular aspects could localize the prolapsed scallop (s) and chordae. The RT3D-TTE findings in 3 patient underwent MV surgery were similar to that obtained by intraoperative 2D-TEE and guided towards the proper surgical techniques of repair.

#### Aortic valve (AV)

Assessment of AV cusps, commissures and area was obtained in 35 patients (10 cases with rheumatic AV stenosis and 25 cases with congenital malformed AV). Good visualization was achieved in 31 patients (88%) by RT3D-TTE while, achieved in 22 patients (62%) by 2D-TTE. Visualization of AV cusps morphology (number, thickness and calcification) and measurements of AV area by planimetry were comparable for both 2D-TTE and RT3D-TTE. In 10 patients with rheumatic AV stenosis, measurement of AV area by planimetery was obtained in 80% and 46% of patients by RT3D-TTE and 2D-TTE respectively. The RT3D-TEE measurement of AV area was correlated well with the 2D-TTE measurement by continuity equation in all cases (r = 0.89; *p *< 0.0001).

**Figure 3:**
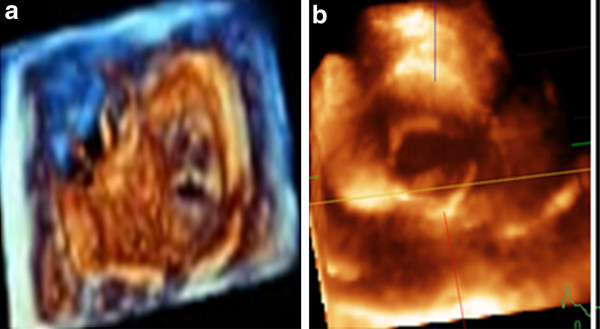
**Morphology of malformed aortic valve cusps: Tricuspid (A) and Bicuspid (B)**.

Among the 25 patients with malformed AV, the valve morphology was bicuspid in 19 patients, quadricuspid in 2 patients, tricuspid in 3 patients and unicuspid in 1 patient (Figure 3). RT3D-TTE and 2D-TTE measurements of AV annulus and LV outflow tract were well correlated (r = 0.85; *p *< 0.001) but the RT3D-TTE measurements of aortic annulus and LVOT diameters were significantly larger than that obtained by 2D-TTE (2.05 ± 0.7 cm and 2.5 ± 0.86 vs. 1.94 ± 0.67 and 1.98 ± 0.74; *p *< 0.01). Measurement of AV area by planimetery could be obtained in 76% and 44% of patients by RT3D-TTE and 2D-TTE respectively. In the remaining 24% of cases the AV area could not be obtained by RT3D-TTE due to heavy calcification. RT3D-TEE measurement of AV area correlated well with the measurement obtained by continuity equation in all cases (r = 0.90; *p *< 0.0001).

#### Tricuspid valve (TV)

**Figure 4:**
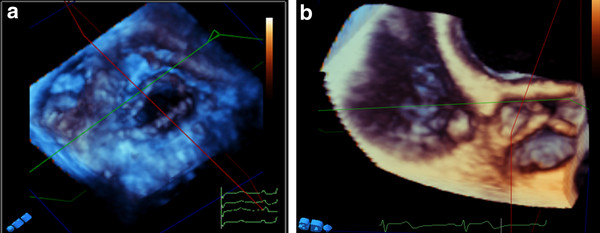
**Enface view of tricuspid valve (A) and pulmonary valve (B) showing all valve leaflets and commissures**.

RT3D-TTE assessment of TV was performed in 32 patients. 2D-TTE examination of those patients demonstrated that 10 patients had normal right- side heart, 7 with pulmonary hypertension, 10 with rheumatic TV involvement, and 5 with dilated cardiomyopathy involving RV. Adequate visualization of the three TV leaflets in the en face view was obtained in 26 patients (81%), while one of the TV leaflets was missed in the remaining 6 patients (19%). The RT3D enface view helped in identification of all TV leaflets in addition to the assessment of leaflets mobility, thickness and calcification and viewing the commissures (Figure 4A). The mechanism of tricuspid regurgitation could be identified in 20 patients as annular dilatation in 10 patients, leaflets thickening and restriction in 4 patients and mixed in 6 patients. In 6 patients with rheumatic tricuspid stenosis, measurement of TV area by planimetery could be obtained with good interobserver agreement.

#### Pulmonary valve (PV)

RT3D-TTE assessment of PV could be performed in 5 patients with an established diagnosis of pulmonary stenosis. RT3D-TTE short axis view could visualize the abnormalities of PV three cusps through analysis of their thickness, mobility and commissures (Figure 4B). Measurement of PV annulus (area and diameter) could be obtained in all patients while PV area by planimetery was obtained in three of them.

#### Prosthetic heart valves and ring

**Figure 5:**
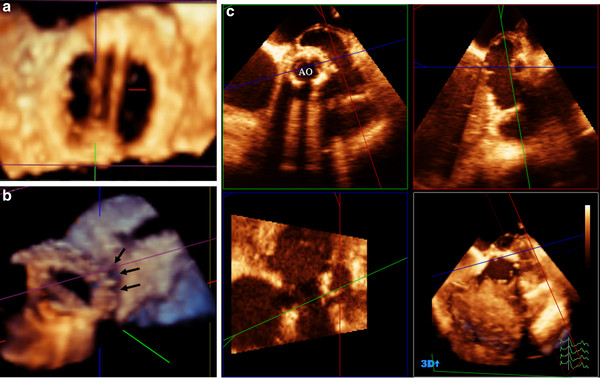
**(A) Morphology of bi-leaflet metallic prosthetic valve in mitral position, (B) bioprosthetic valve with loose attachment to annulus that led to paravalvular leakage (arrows), and (C) Quadscreen of paraaortic abscess due to infective endocarditis of bioprosthetic valve**.

Thirty patients with previous valve surgery were studied (10 bioprosthetic aortic valve, 9 bioprosthetic mitral valve, 3 metallic prosthetic valve and 8 C-rings). RT3D-TTE was performed for the assessment of valve function and to exclude infective endocarditis. RT3D-TTE was of great value in studying the valve mobility, calcification and detection of either vegetation or thrombus. RT3D-TTE with color was performed to assess valvular regurgitation and presence of paravalvular leakage. In every patient, adequate visualization of prosthetic valves and rings was achieved. Well functioning bioprosthetic valve was detected in 7 patients (4 in mitral position and 3 in aortic position). In 13 patients with high clinical suspicion of infective endocarditis, RT3D-TTE could clearly delineate the vegetations (size, site, attachment and number) in 6 patients and para-aortic abscesses (size, site, extension, wall thickness and points of communication) in 7 patients with bioprosthetic aortic valve. RT3D-TTE had additional value for complete definition of paravalvular leakage (site, size and extension) in 6 patients (Figure 5).

### Septal defects (ASD and VSD)

**Figure 6:**
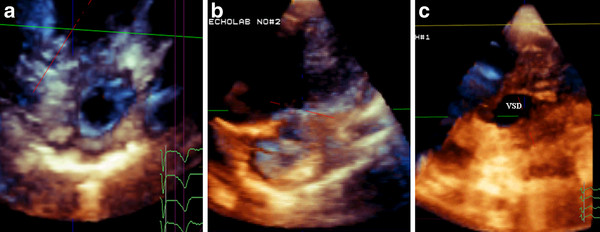
**Enface view of ASD before (A) and after (B) device closure, and muscular VSD(C)**.

A total of 30 cases with suspicious and/or definitive diagnosis of septal defects by 2D-TTE were evaluated by RT3D-TTE. RT3D-TTE could rule out septal defects in three cases and proved the diagnosis of ASD in 18 cases, patent foramen ovale in three cases, and VSD in 6 cases. Through the RT3D-TTE enface view; a comprehensive assessment of septal defects with its relation to adjacent structures was obtained. In addition, enface view allowed accurate measurements of the defect size and rims to assess the suitability for device closure (Figure 6). 14 patients with ASD underwent percutaneous closure with Amplatzer device and 4 patients underwent surgical closure. The RT3D-TTE measurement of defect size showed excellent correlation with that obtained at surgery and by balloon occlusive diameter (r = 0.930, *p *≤ 0.0001). In patients with VSD, two patients underwent device closure and four patients underwent surgical closure. Post-procedure RT3D-TTE assessment of the 16 patients who underwent device implantation was valuable in the assessment of device position and ruling out residual shunt.

### Intra-cardiac thrombi

**Figure 7:**
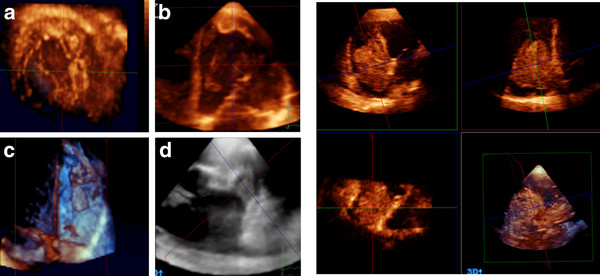
**Left panel showing different examples of intracardiac thrombi into LV (A, B), RV(C), and LA appendage (D)**. Right panel showing quadscreen of RA myxoma.

Forty five intracardiac thrombi were detected by both 2D-TTE and RT3D-TTE (34 in the LV, 6 in the LA, 4 in LAA, 4 in the RA, and 2 in the RV). RT3D-TTE could detect 7 additional thrombi in 5 patients (3 in the LAA and 4 in the LV apex). Examination of LV and LA was obtained from apical views. Assessment of LAA was performed through modification of short axis at aortic valve level, apical 4 and apical 2-chamers views. RT3D-TTE images provided a comprehensive description of the different shapes of the thrombi (horse-shoe shape, bilobed, multi-lobulated, oval, and irregular), attachment to the cardiac wall and their mobility. Multiple cut sections at different levels helped in identification of the thrombus consistency, presence of degeneration and/or calcification in chronic organized thrombi (Figure 7-left panel).

Both 2D-TTE and RT3D-TTE techniques showed good correlation for the measurement of the maximum diameter of the thrombus (R = 0.89, *P *< 0.001). However, RT3D-TTE measurements were larger than 2D-TTE (27.6 ± 9.9 mm vs. 22.4 ± 7.1 mm; *p *< 0.0001). Volume quantification of the thrombi was only obtained by RT3D-TTE (9.4 ± 6.7 mm^3^). Transesophageal 2DE was performed for the assessment of 13 thrombi detected by RT3D-TTE (7 inside LAA and 6 inside LA). There was an excellent agreement between both techniques for visualization of thrombi (Kappa: 0.90) with no significant difference between the measurements of the maximum diameter of thrombi (23.6 ± 6.7 mm and 22.4 ± 8.1 mm respectively; *P *= 0.7). RT3D-TTE showed very good interobserver agreement for visualization of LV and LA thrombi (Kappa: 0.81), while it was good for LAA, RA and RV thrombi (Kappa: 0.41). 2D-TTE showed good interobserver agreement for LV thrombi (Kappa: 0.46), and poor agreement for LAA, RA and RV thrombi (Kappa: 0.31). The 2DE and RT3D-TTE measurement of maximum diameter by the 2 observers showed good correlation (R = 0.95; *p *< 0.0001 and R = 0.99; *p *< 0.0001 respectively). According to Bland and Altman method for interobserver agreement, RT3D-TTE measurements showed better agreement (2.88,-1.92) than that of 2DE (5.33,-3.87).

### Intra-cardiac tumors

LA myxoma was detected in three patients and RA myxoma in two patients. Cardiac metastasis was detected inside RA in two cases with advanced hepatoma. In all cases, 2D-TTE could identify the masses location and anatomical relationship with the surrounding structures. RT3D-TTE identified the site of attachment, the extent of the intracardiac tumors, and their influence on valvular function. Volume calculation of intracardiac masses could be obtained only with RT3D-TTE. Surgical view could be obtained by RT3D-TTE through electronically dissect cardiac structures which was very helpful pre-operatively in facilitating surgical planning (Figure 7-right panel).

## Discussion

The current study described our initial experience of RT3D-TTE application (feasibility, data analysis, difficulties and limitations) in 320 patients with different cardiac abnormalities. In addition, our aim extended to standardize the technique in order to facilitate routine use of RT3D-TTE for cardiac assessment. Our initial experience demonstrated that with good image quality (75% of patients), RT3D-TTE is feasible and could provide comprehensive assessment of all cardiac structures that helps in understanding the mechanism of most cardiac abnormalities in adults. The main study findings include:

(i) RT3D-TTE volume quantification of LV is feasible and accurate due to elimination of the geometric assumption

(ii) RT3D-TTE could define the surface anatomy of cardiac valves and their relation with surrounding structures. It can be used independently for estimation of stenosis severity and understanding the mechanism of regurgitation.

(iii) RT3D-TTE evaluation of prosthetic valves and rings allows a detailed assessment of prosthesis function which is crucial in decision making.

(iv) RT3D-TTE enface views provides a comprehensive visualization of ASD with better assessment of shape, size, area, rims and number of defects.

(v) RT3D-TTE assessment of intracardiac thrombi and masses is feasible and could provide more valuable information than that obtained with 2D-TTE.

### Quantification of LV volume and function

Multiple studies demonstrated the RT3DE accuracy for the assessment of LV volume and ejection fraction with very good correlation and close agreement with computed tomography and magnetic resonance imaging (MRI) [9-11]. In our study, RT3D-TTE assessment of LV functions was feasible in 86% of cases, while in 14% volume quantification could not be obtained due to inadequate visualization of endocardium. This is in agreement with Tighe et al [12] who described the reliability of RT3D-TTE assessment of LV volume and function in 60% of patients who have good and fair image quality.

RT3D-TTE allowed fast and easier analysis of regional volume throughout the cardiac cycle. This was helpful in calculating the time required to attain minimum regional volume and thus detection of mechanical asynchrony. Many studies described the accuracy, reproducibility and feasibility of RT3DE in defining systolic asynchrony in patients with and without electrical asynchrony [5,13]. In our study, RT3D-TTE was helpful in guiding the placement of the pacing wire and to judge the immediate response to CRT.

Assessment of right ventricular (RV) size, volume and function has been extremely challenging in clinical cardiology due to its complex geometrical crescent shape. RT3D-TTE for RV volumes quantification was shown to be accurate and reproducible as compared to MRI [14]. In our study, we used disc summation method for calculation of RV volume and ejection fraction which was shown to be accurate and comparable to MRI findings [15]. Complete analysis of RV volumes and function was obtained in 55% of our patients. RT3D-TTE measurements were well correlated but significantly underestimated as compared to CMR. In patients with markedly dilated RV, the analysis was incomplete due to inability to include all RV borders within the 3D box. There are only few data studying the accuracy of RT3D-TTE in significantly dilated or dysfunctional RV [16].

### Evaluation of valvular heart disease

#### Mitral Valve

Several studies demonstrated the feasibility of RT3D-TTE in evaluation of mitral valve morphology independently from the window of image acquisition [17,18]. It provides a unique orientation of leaflets, commissures and valve area in a considerable percentage of the population [19]. Our study demonstrated that RT3D-TTE could provide better morphologic description of the MV and measurement of MVA in 20 patients with rheumatic mitral stenosis. It was useful for assessment of outcome post- balloon commissurotomy through clear visualization and proper assessment of commissural splitting.

In agreement with Agricola et al [20], our study showed that the RT3D-TTE assessment of MV apparatus and MV annulus was helpful in identifying the mechanism of mitral regurgitation. The shape and size of regurgitant jets were completely assessed only when the jet was central but eccentric and/or large jets were not depicted due to narrow color sector. In accordance with the previous studies [21,22], the anatomic localization of the prolapsed MV scallop (s) was obtained from the enface RT3D view.

#### Aortic Valve

Imaging of AV from either the transthoracic or transesophageal approach is challenging, probably because of the oblique angle of incidence of the ultrasound beam combined with the thinner leaflets. RT3D-TTE quantification of aortic area by planimetery showed good agreement with the standard 2D-TEE, flow-derived methods, and cardiac catheterization data with the advantage of improved reproducibility [23,24]. In our study, adequate visualization of AV was achieved in 88% by RT3D-TTE vs. 62% by 2D-TTE. Planimetered AV area by RT3D-TTE could be obtained in 67% and 80% of patients with rheumatic and congenital AS respectively while it was less obtained by 2D-TTE (44% and 46% respectively). Measurement of AV area with both RT3D-TTE and 2D-TTE continuity equation were well correlated in all cases (r = 0.89; *p *< 0.0001). RT3D-TTE and 2D-TTE measurements of AV annulus and LV outflow tract were well correlated (r = 0.85; *p *< 0.001 for both). However, the RT3D-TTE measurements were significantly larger than that obtained by 2D-TTE (*p *< 0.01).

#### Tricuspid Valve

Assessment of TV by 2D-TTE is challenging due to the complex geometry of TV. RT3D-TTE overcomes the 2D-TTE limitations and provides a comprehensive morphological and functional assessment in various TV diseases [25]. The utility of RT3D-TTE in the assessment of tricuspid regurgitation was described in various studies [26,27]. In our study, RT3D-TTE analysis of TV annulus (size and function), and leaflets was helpful in understanding the mechanism of tricuspid regurgitation and providing the basis for surgical intervention. In agreement with the previous studies [28,29], RT3D-TTE allowed a detailed assessment of TV leaflets (thickness, mobility, and calcification) as well as measurement of TV area in our patients with TV stenosis.

#### Pulmonary valve

Echocardiographic evaluation of PV anatomy is more difficult than other valves due to poor acoustic access even by 2D-TEE [30]. RT3D-TTE could describe the morphological features of the 3 cusps simultaneously through the en face view only in 60% of patients [31]. This percentage increases in patients with pulmonary stenosis due to increased cusp thickness [32]. In our study, only 5 patients with PV stenosis were examined by RT3D-TTE. It was possible to assess the cusp thickness, commissures and mobility in addition to measurement of PV annulus (area and diameter) and PV area.

#### Prosthetic devices (valves and rings)

Anatomical identification and hemodynamic assessment of valve prosthesis and rings is mainly dependant on 2DE (TTE and TEE). However the sensitivity of both 2D-TTE and 2D-TEE techniques for evaluation of prosthetic valves is lower than with native valves [33]. The initial experience with the use of RT3D-TTE was encouraging but no large studies available [34]. Our study showed that RT3D-TEE could identify the type of prosthesis (rings, bioprosthesis and metallic prosthesis) and assess its function. In mitral regurgitation post MV surgery, RT3D-TTE allowed clear definition of the origin of the regurgitant jet and description of the underlying mechanisms. RT3D-TTE could help in the diagnosis of prosthetic valve infective endocarditis through clear visualization of vegetation, root abscess and valve dehiscence.

### Assessment of adult congenital heart diseases

The literature confirms the clinical utility of RT3D-TTE in evaluation of ASD, VSD, patent foramen ovale and atrioventrcular septal defects comparable and well correlated with the surgical and catheterization findings [35-38]. In our study, the RT3D-TTE enface projection could accurately determine the site, size, number and shape of defects in addition to quantitative recording of septal defect dynamics. This valuable information facilitated the proper decision making either for surgical or catheter based management.

### Assessment of intracardiac thrombi

RT3D-TTE was assumed to be the technique of choice for the assessment of intracardiac masses through the unlimited number of cutting planes in all directions from single full volume 3D data set [39]. In our study, RT3D-TTE allowed a comprehensive description of the different shapes of the thrombi, their attachment to the cardiac wall and their mobility. Multiple 3D cut sections helped in identification of thrombus consistency, presence of degeneration and calcification in organized thrombi.

Clear visualization of LAA thrombus was achieved in (78%) of our patients by RT3D-TTE vs. (33%) by 2D-TTE. In accordance with Karakus et al [40], RT3D-TTE findings of LA and LAA thrombi showed good agreement with 2D-TEE. The accurate assessment of thrombus size is difficult by 2D-TTE due to wide variation of thrombus shape. In our patients, RT3D-TTE volume calculation of intracardiac thrombi was obtained regardless of their shape and orientation. In agreement with previous studies [41,42], Our results described the additional value of RT3D-TTE in the assessment of cardiac masses.

### Study limitation

The study described our initial experience with the RT3D-TTE application. However there are some limitations:

1. RV analysis was obtained using the software designed for LV mainly. The new 3D software dedicated for RV analysis was not available in our echo-lab.

2. The 3D quantification of RV and LV volume and function was compared with cardiac MRI in a small number of patients (25) aiming to verify our 3D measurements by the Q lab software. However, the validation of RT3DE was intensively validated in previous studies and this is beyond the target of current study.

## Conclusions

RT3D-TTE provided comprehensive anatomical and functional assessment of many cardiac disorders that can help in confirming the diagnosis, elucidation of the disease mechanism and planning proper management. This will encourage its routine use in daily practice.

## Abbreviation

AV: Aortic valve; ASD: Atrial septal defect; CRT: Cardiac resynchronization therapy; EF: Ejection fraction; IVC: Inferior vena cava; LV: Left ventricle; MV: Mitral valve; PV: Pulmonary valve; RT3D-TTE: Real-time three-dimensional transthoracic echocardiography; RV: Right ventricle; TV: Tricuspid valve; 2D-TTE: 2-dimensional transthoracic echocardiography; VSD: Ventricular septal defect.

## Conflict of interests

The authors declare that they have no competing interests.

## Authors' contributions

AAM has made substantial contributions to design the study in addition to data acquisition and analysis. NYFM has been involved in drafting and revising the manuscript. ZASK has been involved in data collection and analysis. AA carried out the magnetic resonance imaging studies CPH has given the final approval of the manuscript to be published. All authors read and approved the final manuscript.
